# Developing Regenerate: A circular economy engagement tool for the assessment of new and existing buildings

**DOI:** 10.1111/jiec.13377

**Published:** 2023-01-06

**Authors:** Charles Gillott, Will Mihkelson, Maud Lanau, Dave Cheshire, Danielle Densley Tingley

**Affiliations:** 1 Department of Civil and Structural Engineering, The University of Sheffield, Mappin Street, S1 3JD Sheffield, UK; 2 Department of Architecture and Civil Engineering, Chalmers University of Technology, Gothenburg, Sweden; 3 AECOM, London, UK

**Keywords:** circular economy, built environment, construction, design tool, adaptability, reuse

## Abstract

The transition toward a circular economy (CE) is key in decarbonizing the built environment. Despite this, knowledge of—and engagement with—CE philosophies remains limited within the construction industry. Discussion with practitioners reveals this to be contributed to by a lack of clarity regarding CE principles, with numerous organizations recommending implementation of differing and sometimes conflicting principles. In addition, a systematic assessment of how building designs consider CE is made difficult by the multiple design areas required to be considered and the large amount of design data required to do so. The absence of a systematic CE assessment causes a lack of comparability across designs, preventing benchmarking of CE practices in building design at present. This paper details the development of Regenerate, a CE engagement tool for the assessment of new and existing buildings, established in an effort to overcome the aforementioned barriers to the adoption of CE within the construction sector. A CE design workflow for the built environment is proposed, comprising four overarching circularity principles (Design for Adaptability; Design for Deconstructability; Circular Material Selection; Resource Efficiency) and contributing design actions. In addition to engaging stakeholders by enabling the assessment of building designs, the tool retrieves key data for further research. Information on completed design actions as well as recycling and waste metrics is collected to facilitate future CE benchmarking. “Bill of materials” data (i.e., material quantities) is also compiled, with this being key in material stock modeling research and embodied carbon benchmarking.

**Supplementary Information:**

The online version of this article (doi:10.1111/jiec.13377) contains supplementary material, which is available to authorized users.

## INTRODUCTION

The construction industry represents 36% of global energy use and 37% of energy‐related carbon emissions, 10% of which result from the manufacture of building materials and products (United Nations Environment Programme, 2021). Built environment stocks (buildings and infrastructure) are developed to meet human socioeconomic needs such as shelter or transportation. In the prevailing paradigm of a linear economy (take–make–dispose), the development of stocks requires extensive primary resource extraction and generates a significant amount of waste. Krausmann et al. (2017) estimated that, in the 20th century, global material extraction for building stocks grew from 7 to 78 Gt/year, while discarded stocks generated 293 Gt of solid waste. The authors calculated human‐made material stock (built environment and machinery) accumulation to have risen 23‐fold in the 20th century and, assuming emerging economies develop their material stocks to industrial levels by 2050, predict material stocks to rise further by up to four times the present levels. These issues relating to material and energy consumption in the built environment will be further exacerbated if the current linear economy paradigm persists.

Material accumulation into long‐lived structures means the built environment represents a material reservoir that, to date, remains largely untapped. These built environment stocks also embody a large amount of carbon emitted in their pre‐use lifecycle stages (i.e., from raw material extraction, manufacture, transportation, and construction). This means built environment stocks ought to stay in use for as long as possible to avoid unnecessary resource extraction and waste generation, and resulting energy use and greenhouse gas (GHG) emissions, associated with replacing them. Keeping built environment stocks in use may be achieved through lifetime extension and material re‐looping, which are key concepts of a circular economy (CE). CE discourse within politics and wider society has gained strength in recent years, contributed to by a worldwide declaration of climate emergency (IPCC, 2021).

In industrialized countries, the vast majority of built environment stocks were constructed long before climate emergency awareness was widespread, and thus without sustainability and CE principles in mind. This has led research communities in industrial ecology and material flow analysis to work intensively on understanding existing stocks through characterization of their patterns and impacts. Topics of material stock analysis include: the dynamics of stock accumulation over time (Fishman et al., 2014; Liu & Müller, 2013; Liu et al., 2013; Müller, 2006) and relationships between stocks, flows, and services (Haberl et al., 2017), as well as the quantification of materials accumulated in buildings and infrastructure (Tanikawa et al., 2015) to better understand secondary resource recovery (Lanau & Liu, 2020; Oezdemir et al., 2017), embodied carbon emissions (Lanau et al., 2021; Mao et al., 2021; Stephan & Athanassiadis, 2017), and end‐of‐life scenarios (Mastrucci et al., 2017; Schiller et al., 2017).

As current stocks and opportunities for material recovery are increasingly understood, future stocks (e.g., new builds) must be designed with CE concepts in mind to maximize their service supply while minimizing whole‐life environmental impacts. Successful examples of CE design in the built environment remain anecdotal however, and the industry‐wide implementation of CE required to achieve decarbonization of the construction sector will not occur until underlying CE concepts are more widely understood (Guerra et al. 2021). This lack of awareness and understanding of CE concepts and strategies in the construction sector poses the question of how CE knowledge may be conveyed—and implementation encouraged—within industry.

In this paper, we present the development of a circular economy engagement tool for the assessment of new and existing buildings, which aims to engage construction industry stakeholders with CE philosophies across all project stages. The structure of the paper is as follows. Section 2 summarizes concepts and sometimes conflicting definitions of CE and highlights the definition of CE adopted in the paper. Strategies and policies to support CE in the built environment are also reviewed, along with circularity indicators and tools currently available for use in the construction industry. Limitations of these are then highlighted, framing aims and objectives of the tool. In Section 3, we present the development of the tool from initial prototype to final release, highlighting development milestones such as stakeholder workshops and ensuing changes, and functionalities added to meet the tool's objectives. In Section 4, we assess and reflect on the tool's delivery of its objectives, as well as further achievements. We also highlight the international applicability of the tool, enabled by international considerations throughout the design process, and the tool's ability to strengthen collaboration between academia and industry practice.

## CIRCULAR ECONOMY IN THE BUILT ENVIRONMENT: STATE OF THE ART

### Defining a circular economy

The first academic discussion on linear versus circular management of resources dates back to 1982 with Stahel's (1982) work on a functional service economy. Already then, Stahel highlighted the importance of increasing the “use‐life of goods” in transitioning to a more sustainable society, and theorized a self‐replenishing economic system with different levels of replenishing loop (reuse, repair, reconditioning, and recycling). CE concepts were also part of other schools of thought, including the field of Industrial Ecology theorized by Lifset and Graedel (2002), the Cradle‐to‐Cradle design philosophy of McDonough and Braungart (2002), and Natural Capitalism by Hawken et al. (1999). Thirty years after Stahel's functional service economy, the Ellen MacArthur Foundation (EMF) was launched, with the mission to accelerate the transition to a CE. The EMF's butterfly diagram (Ellen MacArthur Foundation, 2013), illustrating the flows of technical and biological material through the “value circle,” has been widely adopted and adapted for use across research and practice. EMF's value circle includes several cascading loops through the product value chain, with the aim of recirculating resources already present in our socioeconomic system.

The overarching concepts of CE emphasize the need to keep materials in the socioeconomic system for as long as possible, at their highest possible value. However, a number of scholars (Bocken et al., 2016; Stahel & Clift, 2016) noticed a tendency to focus on re‐looping of flows while overlooking the role of material stocks. These material stocks are key components of our socioeconomic metabolism; they provide services (e.g., shelter, transportation infrastructures, access to energy and water) (Haberl et al., 2017) and are intrinsically linked to the type and amount of material and energy required to supply other flow‐based services (e.g., the amount of energy needed to heat a house depends on its thermal efficiency; dictated by the materials from which it is built).

In economies where high quantities of stocks have accumulated, lifetime extension is a critical strategy for sustainability, as advocated by Stahel and Clift (2016) in their concept of a “performance economy” that “goes beyond most interpretations of a ‘circular economy’.” In a performance economy, the focus is on maintaining and improving existing stocks and on maximizing the services they supply to humans, rather than recirculation of material and energy. Nevertheless, the concept of product lifetime extension can still be found in academic literature on CE. For example, Bocken et al. (2016) categorized circular approaches into three key areas: (1) those that slow resource flows through the design of long‐life goods and product life extension (akin to the performance economy described above), (2) those that narrow resource flows through more efficient design, and (3) those that close resource flows through reuse and recycling at end of life.

It is clear that focusing on prolonging stock use and ensuring re‐looping of stocks are highly relevant strategies in the transition to a more sustainable economic model, irrespective of adopted nomenclature. In this article, “CE” is used as an umbrella term encompassing all the above‐mentioned approaches. In the built environment, lifetime extension of buildings is recognized as the most environmentally rewarding of these, followed sequentially by element‐wise reuse, remanufacturing, recycling, and downcycling, with energy recovery and landfilling being the least favorable resource management approaches.

### Strategies and policies to support implementation

In addition to academic work defining CE philosophies, there is a growing body of grey literature outlining the steps necessary to embed CE strategies within building and infrastructure design. This includes work from organizations of varying geographic coverage, including the global (Arup, 2016; World Business Council for Sustainable Development, 2021; The Ellen MacArthur Foundation and Arup, Acharya et al., 2018), international (Circle Economy, Dutch Green Building Council, Metabolic, & SGS Search, 2018; European Commission, 2020b), national (UK Green Building Council (UKGBC), 2021; Holland Circular Hotspot, 2022; Vrije Universiteit Brussel (VUB), 2019), and city level (London Energy Transformation Initiative (LETI), 2020;, Re:London (formerly LWARB), LWARB, 2017).

We highlight here a particularly interesting concept, “building in layers,” mentioned in three guidelines (Cheshire, UKGBC and Arup, Greater London Authority, 2020). Often referred to as “shearing layers,” this concept was first posited by Duffy (1992) and further developed by Brand (1994). They suggested buildings should comprise layers of components with different lifespans: site (eternal); structure (30–300 years); skin (20 years); services (7–15 years); space (3–30 years); stuff (1 day–1 month) (Brand, 1994). This concept is relevant in the CE paradigm as, by organizing components based on their predicted lifespans, components may be replaced or upgraded with minimal impact on surrounding layers. Avoiding unnecessary building modification (e.g., partial demolition) in this way reduces long‐term resource use and waste generation.

**FIGURE 1:**
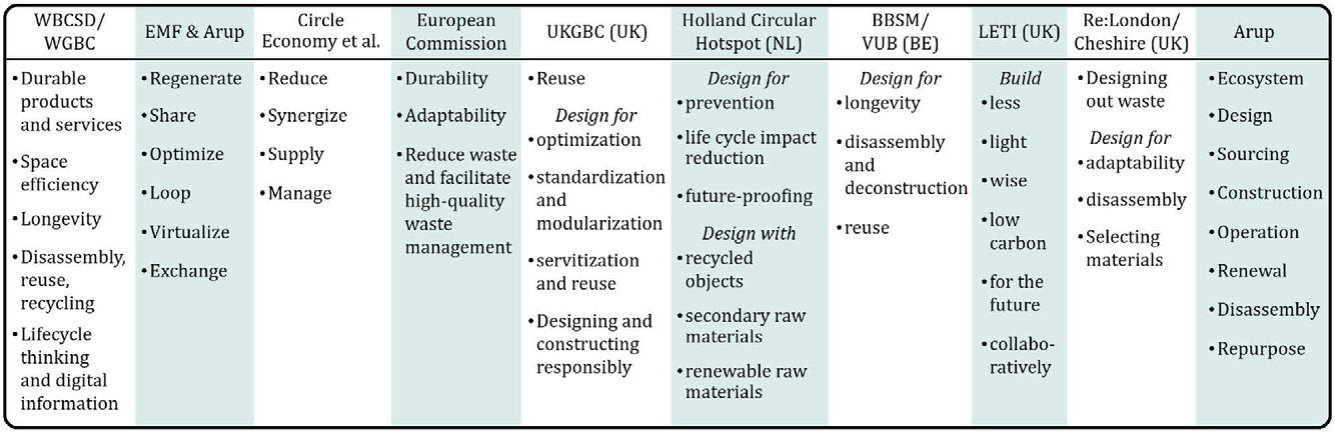
CE design strategies for the built environment as outlined by various international, national, and regional organizations. BBSM, Brusselian built environment, source of new materials; DGBC, Dutch Green Building Council; EMF, Ellen MacArthur Foundation; LETI, London Energy Transformation Initiative; UKGBC, United Kingdom Green Building Council; VUB, Vrije Universiteit Brussel; WBCSD, World Business Council for Sustainable Development; WGBC, World Green Building Council.

Still, while work from aforementioned organizations generally follows core themes of CE and aims to ensure effective integration within projects, summarizing their key design concepts (Figure 1) highlights that currently available CE strategies are numerous, inconsistent, overlapping, and often incomplete and/or conflicting. Additionally, the review of grey literature reveals a lack of design decisions and actions that may be taken to implement proposed strategies. The absence of specific actions may explain, at least in part, why circular design practice remains limited despite efforts to promote CE thinking in the construction industry. This includes increasing policy aimed at accelerating the transition toward a CE in the built environment.

Policies supporting implementation of CE principles in the construction industry include target setting, increased awareness of CE design principles, and stakeholders engagement within built environment projects. At the international level, in 2020, the European Commission launched a CE action plan (European Commission, 2020a) outlining an upcoming “EU Strategy for a Sustainable Built Environment.” This strategy will aim to ensure coherence across relevant policy areas and promote CE principles throughout the lifecycle of buildings.

In the United Kingdom, the ambition to move to a CE is reflected in the recent CE Package, which aligns with other strategies such as the 25 Year Environment Plan and the Resources and Waste Strategy (Department of Environment, Food & Rural Affairs, 2018, 2020; Department of Environment Food & Rural Affairs, 2019). The latter also outlines the government's commitment to work alongside organizations such as the Green Construction Board to define and develop a route map toward zero avoidable waste in the construction sector. Recommendations include mandating pre‐demolition waste auditing, and integrating considerations on recycled and reused materials in Building Regulations and the National Planning Policy Framework (The Green Construction Board, 2021).

At the local scale, the Greater London Authority (GLA) has led the way in policy implementation, including setting out waste reduction objectives to ensure high quality, sustainable buildings that consider CE principles. A CE statement is also mandated as part of the planning application for all major schemes within the Greater London area to impel CE considerations and inform design decisions at early project stages (Greater London Authority, 2020).

Although instrumental in raising awareness and promoting CE practices within the built environment, policy falls short of enabling construction professionals to integrate CE effectively on a project‐by‐project basis. This may largely be attributed to a lack of data and current understanding of CE implementation at scale, which makes key strategic indicators and targets difficult to establish, as noted in the indicator framework for monitoring the Resource and Waste Strategy (Department for Environmental Food & Rural Affairs, 2018). A crucial area to consider is thus the measurement of building circularity through project‐level indicators that are both practically quantifiable with available data, and engage industry professionals with relevant CE philosophies.

### Measuring the circular economy: Existing indicators and tools

There are a vast array of CE indicators aimed at quantifying circularity of products and industries. An international review of academic and industrial literature by Saidani et al. (2019) counted 55 CE indicators, while Parchomenko et al. (2019) found 63 metrics categorized into three areas—resource efficiency, material stocks and flows, and product centric indicators. Examples of indicators include a “longevity indicator,” which measures “initial lifetime,” “earned refurbished lifetimes,” and “earned recycled lifetime” (Franklin‐Johnson et al., 2016); and a ‘“reuse potential” indicator, which quantifies the proportion of economically recoverable resource (Park & Chertow, 2014). Both of these, the latter in particular, require input of a reasonable amount of product data. Niero et al. (2019) recommend linking circularity indicators such as a material reutilization scores (developed by Cradle‐to‐Cradle certification) and material circularity indicators (developed by EMF) with life cycle assessment (LCA) approaches via multi‐criteria decision analysis to compare different product solutions in a holistic manner. A wider systems approach is taken by Pauliuk (2018), who recommends the use of material flow analysis to understand material stocks and flows, and the service they provide. This data‐driven approach has the benefit of being applicable at multiple scales, thus enabling an understanding of CE of businesses, cities, or nations.

Whilst many of these indicators may be applied to the built environment, none were developed with this specific application in mind. This hinders the assessment of buildings, whose circularity is particularly challenging to assess. Rahla at al. (2019) point out the complexity of buildings as problematic for circularity analysis, as buildings contain many different components of varying lifespans, with design strategies being applicable at both building and component levels. Additional challenges include a high data demand for modeling, and potential ambiguity in weight and scoring. Because of these challenges, recent years have seen the release of a number of tools and software add‐ons facilitating the circularity measurement of buildings.

The BAMB Horizon2020 project (involving 15 partners in seven European countries) addresses some of the aforementioned challenges by developing a proof‐of‐concept prototype that can be integrated with building information models (BAMB, 2016). The assessment focuses on relative reuse potential as a key metric, aiming to encourage buildings that can be deconstructed for direct component reuse. This may then be combined to more data‐intensive environmental and economic evaluations to highlight potential benefits. The requirement for large volumes of data and detailed building designs represent significant drawbacks of this approach, alongside its consideration of only the reuse aspect of CE.

The building circularity add‐on for One Click LCA software (One Click LCA, n.d.), used in over 100 countries, offers an alternative measure of CE using a bill‐of‐materials approach. This takes several key data inputs, including quantity (volumes or mass) of each material within a building, the share of these that are renewable, reused, and recycled, and each materials’ proposed end‐of‐life process (e.g., to be reused, recycled, downcycled, or sent to landfill). A circularity rating is then calculated for each material and the entire building using percentages of material derived from non‐virgin sources and redirected from landfill. An arbitrary weighting process where downcycling is suggested to be 50% as circular as recycling or reuse is employed in this step. Opportunity to indicate for which materials disassembly and adaptability have been considered is also provided, although this functionality has no subsequent effect on the circularity rating, limiting measurement of CE to resource extraction and waste generation reduction. The add‐on's requirement for material quantities, which may only be obtained through detailed design, again means that it is data intense and precludes its use in initial project stages.

### Aims and objectives of the tool

Review of the above literature highlights barriers to the adoption of CE in: conflicting design strategies; a lack of support in policy; limited measurement methods; and a resulting absence of benchmarks for CE practice in the built environment. In this paper, we present the development of Regenerate, a built environment‐specific CE tool that aims to address the above knowledge and implementation gaps through four objectives. Specifically, Regenerate should:

1. Propose a CE design workflow for the built environment: collating and refining existing CE design strategies, formulating specific design actions contributing to these, and recommending an order in which these should be considered.
2. Engage a range of construction sector stakeholders in the circular economy throughout project stages.
3. Provide a data‐light means of assessing different types of buildings that considers all aspects of CE and may be applied across project stages.
4. Generate an initial indication of poor, standard, and good CE practice in building design and enable further exploration of CE benchmarking.

## DEVELOPMENT OF A CIRCULAR ECONOMY ENGAGEMENT AND ASSESSMENT TOOL

**FIGURE 2:**
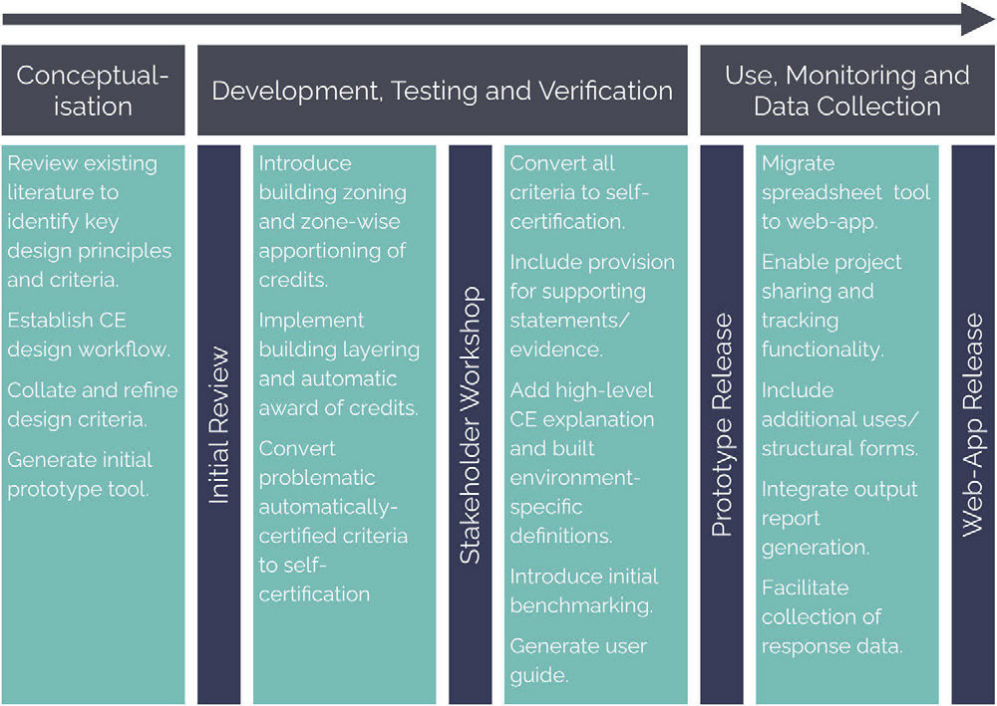
Overview of the tool development process, outlining key stages (grey), actions (teal), and milestones (blue)

The development of Regenerate (Figure 2) consisted of three stages: (1) initial conceptualization of a prototype tool, (2) tool development, testing and verification, and (3) the use, monitoring and collection of data following the tool's release. Each stage is discussed below, spanning four key milestones, two distinct review processes, and two Regenerate releases (prototype and web‐based versions). The chronological presentation of tool's development is a deliberate choice, offering the best opportunities to present and reflect on the outcome of each milestone such as changes and additional features implemented to ensure delivery of the tool's objectives (Section 2.4).

### Tool conceptualization

#### Development of a CE design workflow for the built environment

**FIGURE 3:**
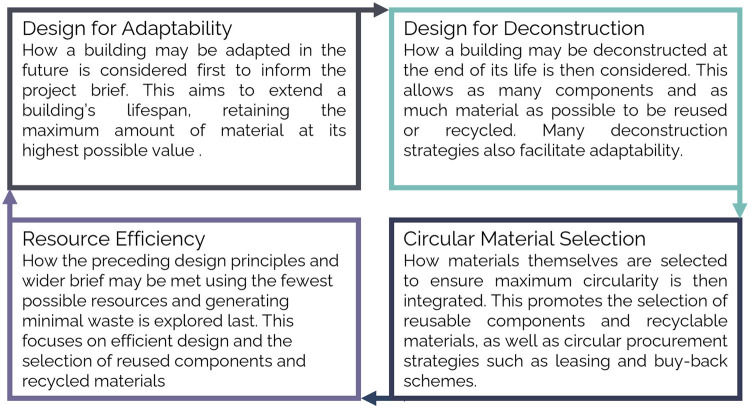
Proposed CE design workflow for the built environment, providing details of each principle, and justification of their order of consideration

In line with objectives in Section 1.4, the first step was to review and refine existing literature on CE strategies into a design workflow for the built environment. This led to the formulation of four high‐level circularity principles: Design for Adaptability, Design for Deconstruction, Circular Materials Selection, and Resource Efficiency (see Figure 3 for definitions). These were developed to maximize coverage of built environment CE design strategies whilst minimizing overlap across principles. Next, in line with Cheshire's (2016) built environment hierarchy, the CE design workflow (Figure 3) was constructed, highlighting the order in which developed principles should be considered to maximize circularity. The workflow thus prioritizes extension of building lifespans (through adaptability) before ensuring the value and reusability of constituent materials is maximized (through deconstructability and circular material specification), and demand for virgin material is reduced (through resource efficiency strategies).

#### Initial prototype generation

**FIGURE 4:**
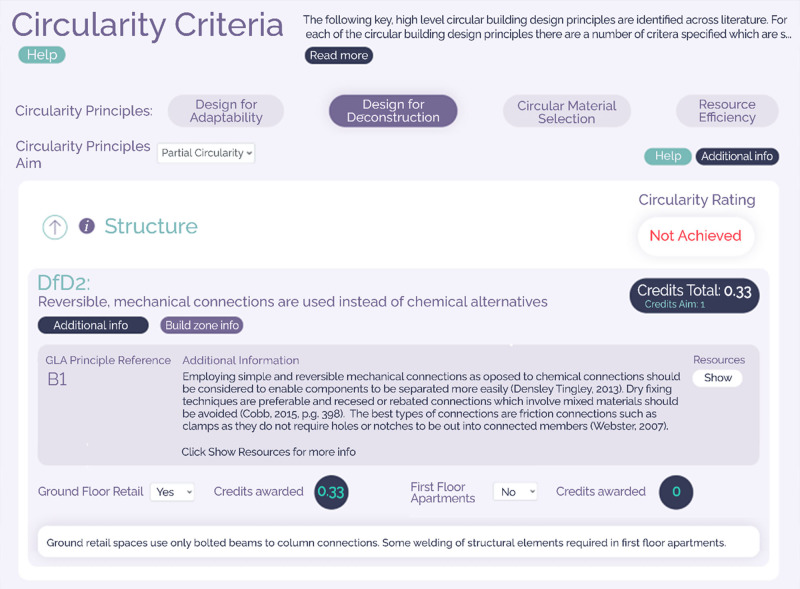
Screenshot of the web‐based application (slightly adapted to increase readability), showing an example circularity criterion, supporting information, and containing CE principle and building layer

**FIGURE 5:**
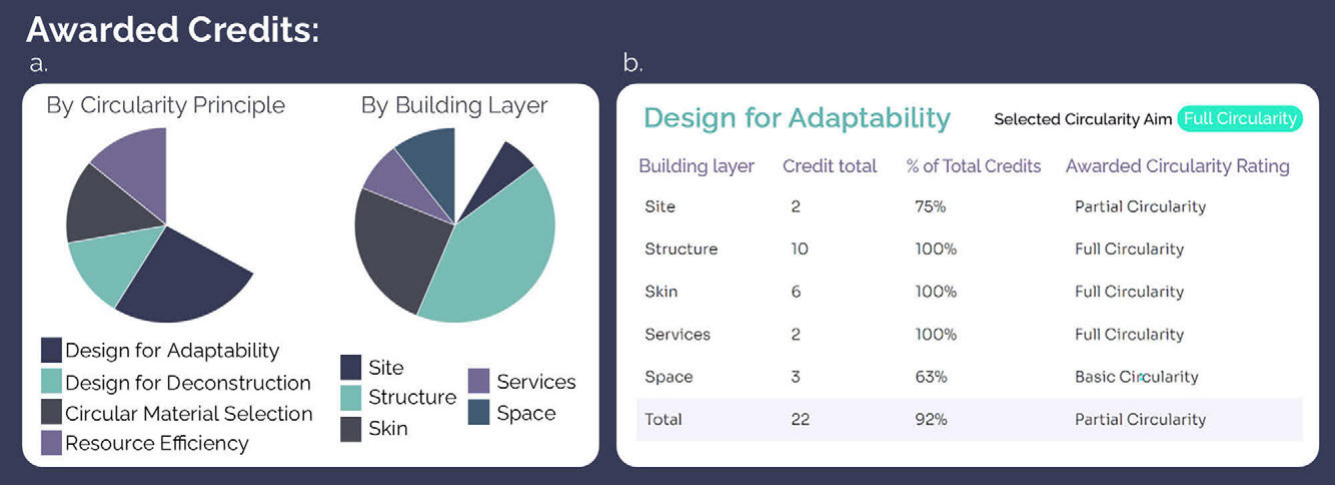
(a) Graphical summary of the credits obtained within each circularity principle and building layer. (b) Tabulated summary of the number of circularity criteria attained within each building layer showing the percentage of selected circularity aim and total credits this represents (slightly adapted to increase readability)

Within the adopted principles, a set of 45 specific design actions for which compliance may be evidenced were developed. These so‐called circularity criteria were synthesized from academic and industrial literature before being reviewed, tailored to the context of the built environment, and assigned to the principle and building layer(s) to which they pertain. An example of one such criteria is shown in Figure 4 (for visualization purposes, Figures 4 and 5 are extracted from the Regenerate web‐app, which is discussed further in Section 2.3). It should be noted here that whilst fulfilling a criterion may assist in the delivery of multiple principles (e.g., reversible connections enable both adaptability and deconstructability), criteria only appear in the principle to which they most closely relate. A criterion may however appear across multiple layers within a principle, for example, both structure and skin may have reversible connections. This results in a total of 86 attainable criteria across all circularity principles and building layers (Table Table 1). A complete list of adopted circularity criteria may be found in the supporting information.

**TABLE 1:** Number of circularity criteria within each circular design principle and building layer

Building layer	Circularity principle				Total
	Design for adaptability	Design for deconstruction	Circular material selection	Resource efficiency	
Site	2	1	1	4	8
Structure	10	5	7	4	26
Skin	6	5	6	3	20
Services	2	4	6	3	15
Space	4	4	6	3	17
Total	24	19	26	17	86

In this initial prototype, a building's compliance with each circularity criterion was assessable via a self‐certification response (i.e., yes or no) or, where possible, automatically using codes of practice, design guidance, and user‐inputted values. One such automatically assessed criterion was “floor loading design criteria allows for flexibility and future change of use,” for which the tool determined compliance by comparing a user‐inputted loading value (in kN/m^2^) with that recommended in Eurocodes for the stated building use. Where a building complies with a circularity criterion, a *circularity credit* (assigned a value of 1) is awarded. Awarded credits are then summed across the entire project into a *circularity overview*, presenting attained credits disaggregated by circularity principle and building layer in both a graphical and tabulated form (Figure 5).

In addition to consideration of circular design practices, Regenerate also promotes material‐level circularity through collection of material quantity and recycling and waste data. A bill of materials style reporting form is provided through which users can input element‐wise material quantities and associated embodied carbon values. The percentage of each element that is from reused sources, and the proportions that are likely to be reusable and recyclable at the end of the building's life, may also be specified. Recycling and waste reporting forms collect waste quantities (weight) for construction, demolition, and excavation works, as well as the percentages of this that are reused and recycled on‐ and off‐site. Both bill of materials and recycling and waste reporting forms are consistent with those required by GLA CE statements (Greater London Authority, 2020). In further consistency with GLA CE statement guidance, the tool recommends a pair of high‐level strategic approaches through which residual value of existing materials and future value of new materials may be maximized. These consider the specific context of the development under consideration (e.g., its permanency and the presence of existing structures or materials on site) to indicate the most appropriate approach to maximize circularity.

### Tool development and verification

#### Initial review

The initial prototype was reviewed in a dual‐moderator focus group attended by four chartered industry professionals. After introducing the rationale and aims behind the tool development, the purposes of the workshop were highlighted, that is, to identify additional requirements of the tool and highlight issues with its current form. A specific focus was set on ensuring proposed criteria and input requirements were compatible with data available to an engineer at the design stage of a project. The dual moderator setting allowed one moderator to run the focus group through the tool, while the second moderator took note of all comments and feedback and ensured all discussion topics were covered. The key results from this initial review are summarized below, together with ensuing improvements to the tool. For a complete list of comments made in the initial review process please see supporting information.

- Stakeholders indicated the need for the tool to be able to capture complex project types such as mixed‐use schemes, that is, those with a combination of new‐build and refurbishment areas. Thus, concepts of building zoning and layering were introduced. Building zones allow a development to be split up into discrete areas with a unique combination of use type, structural form and development type (i.e., new‐build/refurbishment). The circularity of each zone is then assessed separately, necessitating the splitting of circularity credits into sub‐units. Gross floor areas of each building zone are used for this purpose, with a single credit being apportioned based upon the percentage of the development each zone represents. This enables consideration of complex developments and prevents indication of circularity based upon compliance within only a small area of a development.
- The need to capture complex adaptive‐reuse projects (e.g., facade retention and foundation reuse) was also indicated. The tool was thus adapted to require users to specify which building layers are to be retained within each building zone. This functionality allows different types of adaptive‐reuse projects to be assessed, with non‐applicable criteria (i.e., for retained layers) being omitted and maximum credit score automatically awarded. Such scoring is based upon the notion that retaining any given layer is the most circular strategy available, thereby promoting the retaining of materials at their highest possible value.
- Practitioners also highlighted the unlikely availability of some design information required for automatically certified criteria (e.g., assumed loading) at early project stages. This would preclude the use of Regenerate pre‐design, contradicting its objective to provide a data‐light alternative to existing post‐design assessment tools (Section 1.4). As such, design data inputs were replaced with self‐certification responses for each problematic criteria. To combat potential risk for exploitation of the tool for false claims of CE compliance, additional guidance was included to define conditions with which responses to self‐certification criteria are to be interpreted within the tool. For example, during pre‐design stages, Regenerate states that user inputs must represent a genuine statement of intention, with these being required to be informed by results from concept and detailed designs as the project progresses.

#### Stakeholder workshop

After integration of the aforementioned functionalities, the generated prototype was reviewed in a 3‐h, multi‐disciplinary workshop with 15 stakeholders from across the construction industry. Stakeholders included those in managerial positions (e.g., head of sustainability and sustainability manager), engineers (civil, structural, and architectural), consultants, and architects. These represented 14 organizations whose core business activities are in design and construction of buildings, including real estate developers, sustainability consultancies, construction consultancies, contractor firms, and architecture practices. This diversity of skills was key to ensure meaningful insights on all aspects of the tool, including its functionalities, ability to assess different types of projects across design stages, usability by different stakeholders, and validity of its assessment outputs.

Another key aspect to the workshop was the variety of proposed activities, designed to maximize opportunities for, and different types of, feedback. After a brief introduction and walkthrough of the tool, initial comments were gathered. Attendees were then split into two groups to assess a mock project with the tool, while feedback and comments were encouraged and gathered by moderators. Following this, each attendee was required to fill out a survey, offering opportunity to indicate which circularity criteria they believed to correspond to the award of a “basic,” “partial,” or “full” circularity rating. Finally, a last round of general discussion and feedback was held. Key feedback gathered from stakeholders is summarized below, together with ensuing improvements to the tool. For a complete list of comments made in the initial review process please see supporting information.

- Stakeholders agreed that self‐certification criteria were most suitable in all cases, resulting from varying availability of design data across project stages, stakeholder roles, and seniority levels. Stakeholders perceived the potential risk of users exploiting the self‐certification nature of the tool as minimal, but suggested an additional mitigating strategy, namely requirement of evidence for each instance of criteria compliance. As such, all criteria were reformatted to be self‐certified, but opportunity to provide a supporting statement to justify (non‐) compliance with each criterion was integrated. Input of supporting statements remains optional to allow for expression of intention, and thus use of the tool, at early design stages.
- Stakeholders also pointed to a lack of understanding regarding high‐level CE philosophies, and confusion surrounding the built environment specific principles and criteria within the tool. Introductory sections and definitions of key terms were thus integrated, including additional hyperlinks providing further information where required. Criteria were also edited to contain less specialist vocabulary and to consistently follow the same positive statement‐style structure.
- Stakeholders highlighted that functionality of the prototype was not apparent upon opening the tool, resulting in an accompanying user guide document being generated. This was subsequently superseded by a supporting website (Urban Flows Observatory, 2021) following the tool's implementation as a web‐based application (Section 3.3).
- Stakeholders agreed that any weighting of criteria based upon their relative impact would be arbitrary as a result of the currently limited understanding of best practice. Credit weighting was therefore deemed out of current scope of work as a key objective of the tool is to engage stakeholders in circular design. Nevertheless, the importance of future CE benchmarking was noted by stakeholders, and further work is planned in this direction (Section 4.2).
- Stakeholder survey responses were collated and a circularity rating for each criterion established to provide an initial indication of which criteria should be prioritized in all cases (i.e., “basic” criteria), and targeted in projects striving for exceptional (i.e., “full”) circularity. In addition to acting as a target for users, this three‐level rating system has the advantage of acting as an initial benchmark against which projects may be measured.

#### Project testing

Following implementation of these changes, the tool was tested on a live new‐build project at the concept design stage. The tool was used within project meetings under observation and occasional supervision of the authors. Live and follow‐up feedback effectively verified achievement of the three first objectives of the tool (Section 1.4). Indeed, content, features, and design of the tool (Objective 1) were observed to engage multiple stakeholders in CE design (Objective 2) whilst providing an indication of building circularity (Objective 3).

### Use, monitoring, and data collection

The final prototype version of Regenerate was released at a launch event on March 25, 2020, attended by circa 70 construction sector stakeholders. At this point, the final spreadsheet version of the tool was made available as a free download, with uptake reaching over 520 downloads. Workshop sessions were also held with advocacy organizations and construction companies, with over 200 practitioners attending in total. From these industry workshops and continual monitoring of wider uptake and feedback, the efficacy of the tool in engaging stakeholders and providing a means of assessment was evaluated and improved in further iterations of the tool.

Regenerate is now implemented as a web‐based application (Regenerate, 2021) enhancing its usability and introducing a number of additional functionalities offered by the web‐based nature of the tool. This version of Regenerate enables remote project sharing to allow design team members to work collaboratively. Automated collection of attained criteria, bill of materials, and recycling and waste data for each project is also facilitated. A supporting website was released alongside the web‐based version of Regenerate, offering information and support to users and acting as a repository for key CE literature used in the development of, and cited within, the tool (Urban Flows Observatory, 2021).

## DISCUSSION AND CONCLUSION

### Aim and objectives: Delivery and reflections

Regenerate meets each of the objectives set out in Section 1.4. The CE design workflow detailed in Figure 2 collates and refines existing evidence on CE design for the built environment, expanding this to propose the order in which design strategies should be implemented and prioritized to maximize circularity. The tool works to engage multiple construction stakeholders in CE by being applicable across professions and project stages, evidenced by its uptake within industry. This is facilitated by its data‐light means of self‐certified assessment, which goes beyond existing tools to consider all aspects of CE across project stages. The three‐level criteria rating system—developed in place of a credit weighing–acts as an initial benchmark against which projects may be measured. This allows implementation of CE to be measured and assessed across the construction industry from the point of launch of the tool, rather than following collection of a primary set of inputted projects. In addition to these aims, use of the tool during the project testing phase proved to enhance circularity of the design under consideration, thus going beyond sole engagement of stakeholders and demonstrating relevance of an early design stage CE tool for the industry. In recognition of this, Regenerate was included as a recommended practitioner resource in a COP26 parallel session on circularity in the built environment (Ellen MacArthur Foundation, ReLondon, & Zero Waste Scotland, 2021).

As the literature review showed CE knowledge in the industry to be lacking globally, the key objectives of Regenerate remain relevant at an international level. Because of this, although the prototype tool was developed in a UK context, international applicability was ensured throughout its development by avoiding use of UK‐specific construction vocabulary. The tool is also freely accessible online and thus useable irrespective of project location.

### Mutually beneficial collaboration between academia and industry for knowledge and data exchange

The need for stronger engagement between academics and practitioners has been identified as a key component of successful implementation of sustainability strategies (Christ & Burritt, 2019). Indeed, collaboration with industry facilitates knowledge transfer—one of the main objectives of the tool. Furthermore, collaborating with practitioners can also be beneficial for researchers as it provides key insights into current challenges faced by the industry, and can foster relevant research ideas. In the case of Regenerate, an additional benefit is the transfer of data from practitioners to researchers, as the tool automatically collects data relating to attained criteria and, where provided, recycling and waste metrics and bills of material. Such data, notably hard to obtain, is key to several fields of ongoing research.

In time, Regenerate's collection of attained criteria, bill of materials, and recycling and waste data will enable benchmarking of CE practice in the construction sector, and potentially influence future regulation. The commonality with which criteria are attained will allow for generation of more informed and pragmatic circularity aims to supersede existing basic–partial–full scale. These will signify poor, standard, and good practice by comparing the attainment of a given project with the criteria attained by projects on average. There is also scope to introduce weighting across circularity criteria based upon this, with attainment of rarely achieved criteria potentially being rewarded more generously than criteria attained on a large number of projects.

A similar process may also be carried out using bill of material data collected by Regenerate to create poor, standard, and good practice benchmarks for recycled/recyclable and reused/reusable material percentages. These may then be reintegrated as targets within later versions of the tool, with higher recycled/recyclable and reused/reusable material percentages being rewarded more generously to further promote circular practice. Bills of materials are also a key dataset in bottom‐up material stock analysis, and deriving such data from building plans is often time‐consuming for researchers, and remains one of the only ways to derive satisfactory material content in buildings. Here, the tool allows researchers to gather this much needed data, discretized by element or layer, thus helping to populate datasets for material stock studies in the built environment (Heeren & Fishman, 2019). Finally, material quantities may also form the basis of an embodied carbon assessment for each analyzed project. This has scope to utilize carbon intensities from established industry databases or user‐inputted, per‐unit kgCO_2e_ values, though greater difficulty is expected for reused/recycled materials as a result of less readily available carbon intensity data and inconsistencies in their LCA calculation process. In addition to use in academia, such embodied carbon assessments are consistent with growing advocacy for whole‐life carbon assessment, benchmarking, and regulation in industry, as observed in the United Kingdom (Part Z, 2021), United States (Carbon Leadership Forum, 2020), and internationally (World Green Building Council, 2021).

## Supplementary Information

**Supporting Information S1**: This supporting information provides additional information regarding tool development and verification steps (section 3.2 of main text). Firstly, we outline key results from both the internal review and the stakeholder workshop, highlighting feedback that shaped the development of the prototype tool. We then provide an overview of the live case study project used to test the final prototype version of Regenerate, finally outlining the resulting circularity criteria and their extension into current policy within the Greater London area.

## Data Availability

Data sharing not applicable to this article as no datasets were generated or analyzed during the current study.
