# Supplementary material for: Developing Regenerate: A circular economy engagement tool for the assessment of new and existing buildings
Source: J Ind Ecol. 2023 Jan 6;27(2):423–35. doi: 10.1111/jiec.13377 (PMC13083413; doi:10.1111/jiec.13377)
Supplement: Supplementary file 1 — Supporting Information S1: This supporting information provides additional information regarding tool development and verification steps (section 3.2 of main text). Firstly, we outline key results from both the internal review and the stakeholder workshop, highlighting feedback that shaped the development of the prototype tool. We then provide an overview of the live case study project used to test the final prototype version of Regenerate, finally outlining the resulting circularity criteria and their extension into current policy within the Greater London area. [file 44498_2023_2702004_MOESM1_ESM.docx]

**
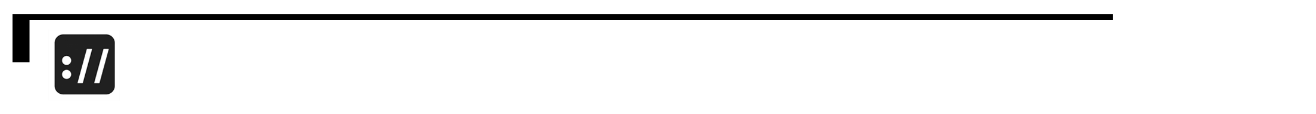
 SUPPORTING INFORMATION FOR:**

Gillott, C., Mihkelson, W., Lanau, M., Cheshire, D., & Densley Tingley, D. Developing Regenerate – a circular economy engagement tool for the assessment of new and existing buildings. *Journal of Industrial Ecology*.


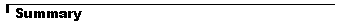
This supporting information provides additional information regarding the tool development and verification (section 3.2 of main text). Firstly, we outline key results from both the internal review and the stakeholder workshop, highlighting feedback that shaped the development of the prototype tool. We then overview the live case study project used to test the final prototype version of Regenerate, finally outlining the resulting circularity criteria and their extension into current policy within the Greater London area.


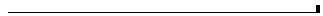


Further information regarding the functionalities of the tool and the application of the tool to projects can be found at the following links:

<https://regenerate.urbanflows.ac.uk/about/>

<https://regenerate.urbanflows.ac.uk/about/user-guide/>

<https://regenerate.urbanflows.ac.uk/resources/>

# Internal review

As outlined in the main text, a dual-moderator setting enabled one moderator to address discussion topics whilst noting feedback and comments, with the other guiding participants through the functions and circularity criteria of the tool. The discussion topics used to guide the internal review related to the application of the tools capabilities within an industry setting to ensure that industry professionals are engaged with the circular economy and are able to apply the tool to projects.

## Bill of materials and recycling and waste forms

Feedback firstly related to the bill of materials and recycling and waste forms which are part of the GLA Circular Economy Statement Guidance. Participants addressed the disaggregation of the bill of materials form, particularly of the structure layer, by element and material and thus evaluated the ability of a practicing engineer to adequately populate the tables at given project stages. Feedback relating to the bill of materials highlighted that structural engineers may be nervous about element-wise disaggregation at concept stage due to increased workload. As quantity surveying and cost analyses increase in confidence through project stages, such disaggregation would be realistic at the end of scheme design (architects drawings at RIBA project stage 2 and engineers at RIBA project stage 3). Supporting information is therefore incorporated to ensure engineers are aware that indicative values may be used at early project stages, with values becoming more accurate as the project progresses. Feedback relating to the recycling and waste forms highlighted that such information may be difficult to input into the tool as contractors are often responsible and carry out such actions. Therefore, broad-brush answers would be more appropriate given the use of a tool by an industry professional in a consultancy setting, where the tool should note this to ensure that engineers engage with the reporting form and consider such information early in the design process. Further feedback on the recycling and waste form suggested that pioneering and standard practice drop down options should be available to the user. This would auto-populate the table based upon intentions and engage users by guiding them to suggested figures which can be amended throughout the project. However, the lack of adequate data in this space limits such information from auto-population, thus the data collection running parallel to the use of the tool in industry is intended to be used to address this suggestion in future iterations.

## Building overview


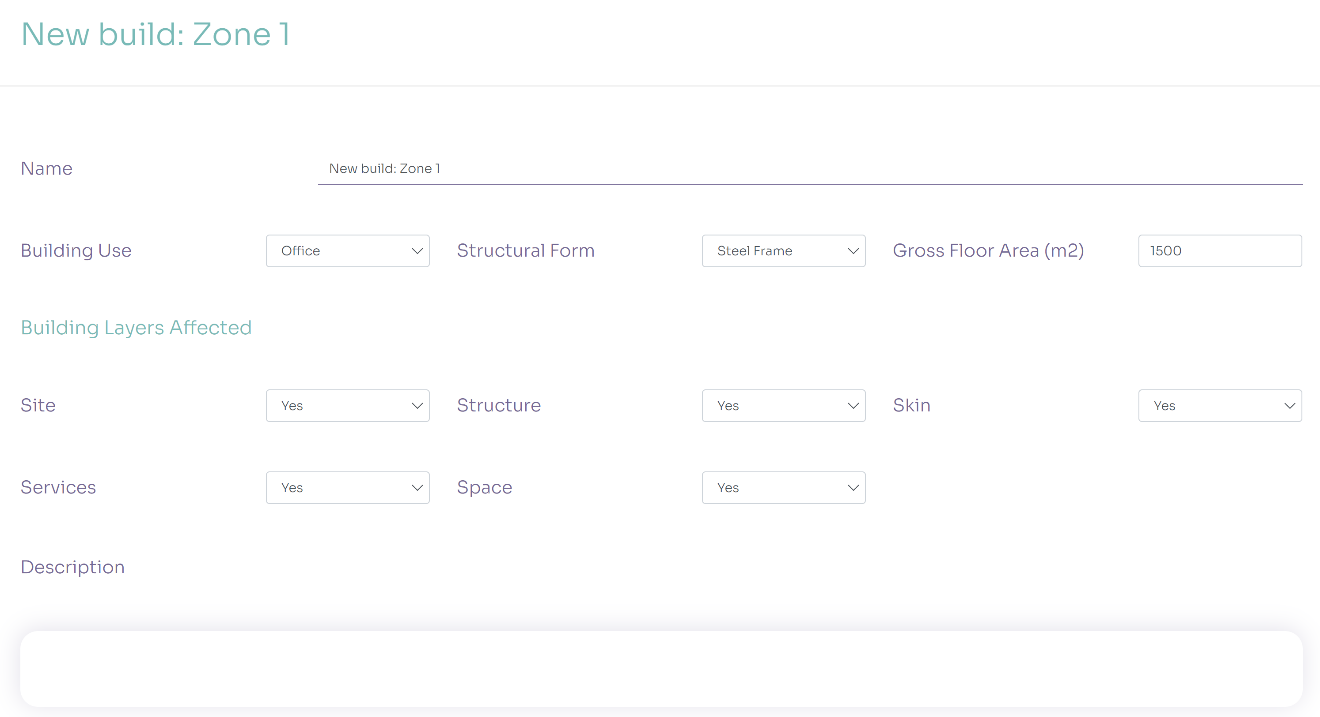

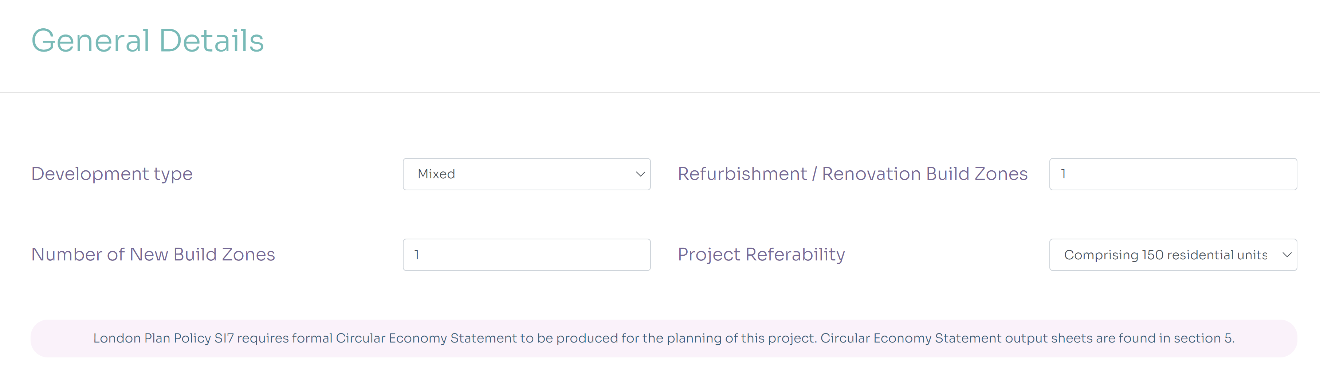
Secondly, feedback relating to the building overview highlighted the inability of the prototype tool to capture a wide range of project types and consequently assess the circularity of projects. These related to projects such as façade retention schemes, reuse of only substructure, and vertical extension, with basement conversion projects popular in London. Participants suggested greater disaggregation of information at the ‘building overview’ stage within the tool. This would indicate which layers are affected for existing buildings or mixed development type, i.e., refurbishment and new build projects, resulting in the introduction of building zoning as described in the main text (section 3.2.1).


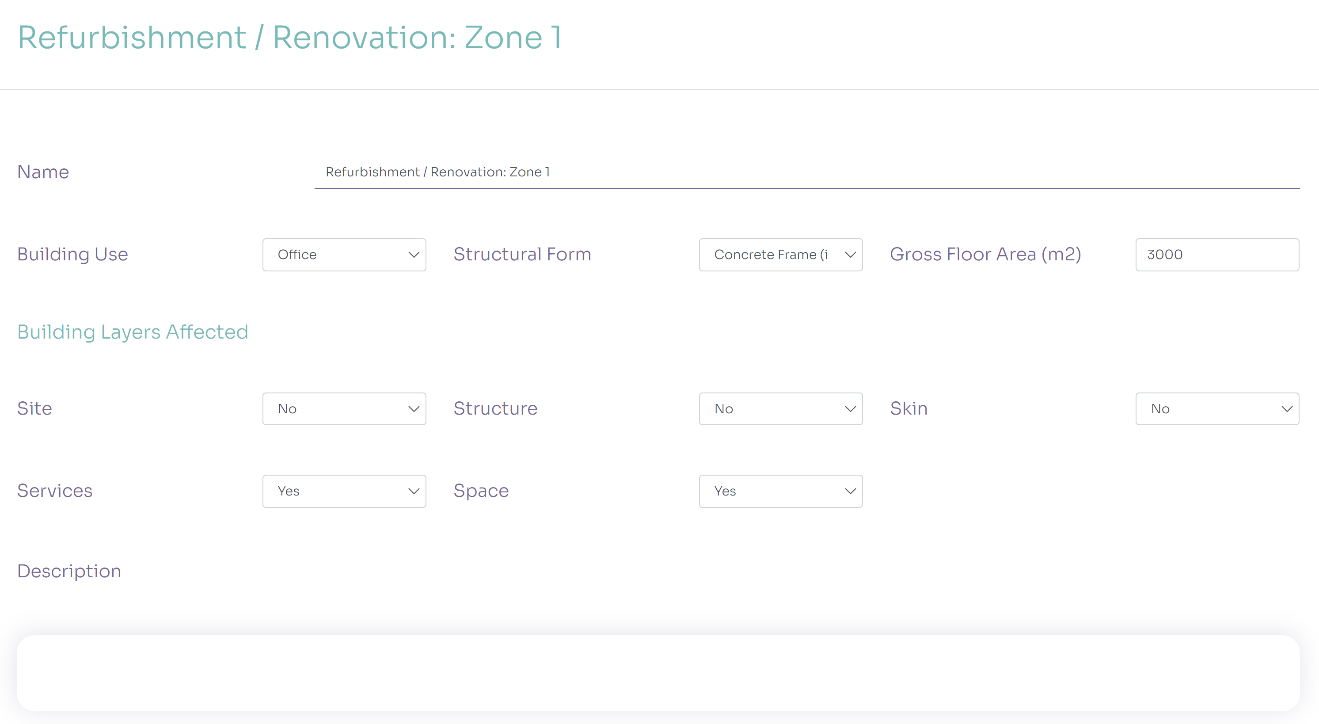


Figure 1: Building overview stage within regenerate, highlighting the incorperation of building layering and mixed used developments. The example demonstrates the ability of the tool to address complex mixed development types by highlighting development type and the layers affected. In this case, the refurbishment scheme relates only to upgrade of services and space layers, whereas the new build scheme affects all layers. The circualrity criteria are then weighted by the relative contribution of gross-floor area to the total for each zone, with criteria relating to unaffected layers being automatically awarded (e.g., in the example above, all circaularity criteria would be awarded to the site, structure, and skin layers in the refurbishment scheme, with credits weighted as new build = 1/3 and refurbishment = 2/3 of the total).

## Circularity criteria

Finally, partipants evaluated the circularity criteria for each circularity principle. Firstly, connection types across layers realting to the deconstructability of the building were discussed. Feedback suggested that users may need more detail regarding the suitability of connection types, whilst the criterion may need to address the use of wet-trades as opposed to the connection types directly which could consequently relate to the input structural form in the building overview stage. Moderators suggested the criterion could be broader, addressing whether connections are reversible and mechanical as opposed to chemical, and was agreed by particpants given adequate additional information to support the self-certiciation of the criterion. An additional criterion was also suggested for the site layer of the resource efficiency ciraculrity principle, addressing whether temporary works are reusable if used on the project. Finally, participants suggested including exemplar projects into each circualrity principle for reference to users.

## Summary

In summary, the internal review process highlighted that greater clarity is required across all stages of the tool to ensure that stakeholders are not overwhelmed by the volume and detail of information required. The result is that pop-ups containing definitions and supporting information relating to the pages, terminology, the approach for populating them across project stages, as well as the circularity criteria themselves are clear and enables timely, and informed decision making. This is supported by self-certication criteria replacing design data inputs for each problematic criteria identified, and a greater disaggregation of project type enabling the tool to capture a wide-range of project types whilst automatically awarding the reuse of building layers in their entirety.

# Stakeholder workshop

After incorporating the previously mentioned feedback the resulting prototype was evaluated in a three-hour multidisciplinary workshop with 15 stakeholders from across the construction industry. The agenda for the stakeholder workshop (Table 1) was used to guide the session and ensure that feedback could be gathered relating to the tools ability to engage stakeholders and assess the circularity of projects. The project used to test the tool in sub-groups was a new-build development at concept design stage constructed from a mix of in-situ reinforced concrete and braced steel frame, providing 16,500m^2^ of study, office, and teaching space across 3 stories and which was expected to be awarded BREEAM ‘outstanding’. Design information pertaining to each building layer and the project as a whole was summarized into an information pack enabling participants to set up and input information into the *regenerate* project during the session. A summary of the feedback relating to the criteria, terminology, tool structure, and usability as well as any general feedback gathered across all sessions is presented in Table 2.

Table 1: Stakeholder workshop agenda

| Sessions | Time | Notes |
| --- | --- | --- |
| Roundtable introduction (all) | ~ 5 mins |  |
| Greater London Authority Circular Economy Statement Guidance Introduction and inception of the tool (all) | ~ 10 mins |  |
| Prototype tool walkthrough (all) | ~ 30 mins | Overview of how to use the tool, it’s intentions and inner workings. Questions and feedback during walkthrough. |
| Testing the tool (in sub-groups) | ~ 60 mins | Brief overview of the case study project to be used. Split participants into two sub-groups and run the project through the tool, mapping what is good, what is missing, what could be improved as well as noting the ease of use. |
| Tool testing debrief (all) | ~ 30 mins | Gather general feedback from participants. |
| Scoring levels/circularity aim discussion (all) | ~ 15 mins | Participants to use the scoring matrix (basic, partial, or full circularity) to indicate which criteria relate to which aim based on the relative difficulty of achievement. |
| Discussion of implications if a refurb projects being used (all) | ~ 20 mins |  |
| Conclusion (all) | ~ 10 mins |  |

Table 3: Feedback and suggestions from participants of the stakeholder workshop

|  | Feedback |
| --- | --- |
| Criteria | Potential future uses must be considered rather than simply assuming the integration of reserve capacity is good in all cases (i.e. overdesign of an office building is not good) |
|  | The ceiling composition is not the only limiting factor in access to services, the wording of the criterion should be changed to reflect this (e.g., ‘is easy access to services provided?’ with note referencing ceiling composition, service placement etc., in additional information). |
|  | There needs to be more consideration of what is already on site – not just in strategic approach |
|  | The benefits of meeting each criterion should be set out in the additional information column (or in a new column). If possible, this should be done using a ‘press release’ metric that will be understood by those with less technical knowledge. – extend this to provide a cost saving? |
|  | Add natural lighting to the natural ventilation criteria (as it was previously)   - - Façade:Depth ratio, Height:depth ratio |
|  | Specific criteria to address demountable partitions. This is particularly relevant in within-use adaptations |
|  | ‘Most common connection type’ – credit score, weld = 0, bolt = 0.5, ?? = 1.0? |
|  | Access to connections is not required for structure layer as all other layers should be removed prior to this (only an issue if a layer is covered by that with a longer renewal period) |
|  | Add a criterion looking at hard or soft landscaping in ‘site’ layer – cut and fill balance may also be placed here |
|  | Refer to recycling AND reuse when referring to the separation of composites |
|  | The declaration of ingredients list should be included as a detail of the material passport criteria rather than as its own criteria |
|  | Potential to link criteria to RIBA plan of work better (potentially note at which stage each criteria must, should and could be filled in?) |
|  | Address offsite construction criteria and reword for each layer (i.e. modular/panelized) |
|  | Prioritize on-site re-use |
|  | Structural engineering adaptability inputs could be made simpler for ease of input |
|  | Add more criteria relating to serviceability limit states on adaptability tab |
|  | Change design for adaptability to design for change to avoid confusion with flexibility etc |
|  | Material passport criteria should be repeated in each layer |
|  | When considering service life include a prompt to suggest that, even when the initial use os only required for a short period, the building be designed with a service life well beyond this for future adaptability |
|  | A definition of material passport should be provided along with guidance on how to complete one |
|  | Criteria within DfD and DfA are too detailed for stage 2 |
|  | Responses to criteria in DfD are often not ‘yes’ or ‘no’ |
|  | If answer is ‘could be’ – how is this followed up? Checked at alter stages? (DfA) |
|  | Add a ‘don’t know’ response so that questions are not left blank at RIBA stages for which the answer is not known |
|  | DFA – cell C40 – what if the building is not naturally ventilated? – far too detailed for RIBA stage 2 |
|  | Provide a list of suggested websites for reused materials etc. |
|  | Add a criterion to capture the completion of a pre-demolition audit? |
|  | Stress how the deconstruction plan must encourage safe practice |
| Terminology | Wherever ‘recycling’ is referenced in any criteria, this should refer to the 3 r’s (reuse, refurbish, recycle). It should also be noted in additional information that users should strive to ensure recirculation along the ‘tightest loop’ in the Ellen MacArthur Foundation diagram. |
|  | Replace ‘minimize use of secondary finishes’ with ‘minimize finishing’ |
|  | Rename ‘envelope’ as ‘skin’ for 5 s’s |
|  | Remove terminology such as ‘as far as reasonably possible’ to get more meaningful responses |
|  | The definition of a deconstruction plan must be provided. What is expected? When should it be completed? |
|  | What constitutes short or long lifespan on the strategic approach tab? |
| Tool structure | Consistency is required across ‘Table 2’ in the GLA and CE principles. These should not be different (this is only an issue due to late integration of table 2) |
|  | Each criterion should appear in the ‘CE principle’ it is most relevant to, with note being made in additional information which other CE principles this also helps address |
|  | Criteria should be disaggregated by layer first (one tab for each layer) and then by ‘CE principle/GLA principle’ next |
|  | Include ‘site’ layer |
|  | ‘Project type’ could be used as with SKA to differentiate GLA requirements from use in other projects |
|  | Criteria should be split up by GLA table 2 rather than CE principles |
|  | ‘Long and short’ in strategic approach could be renamed ‘temporary and permanent’ as with service life in EC |
|  | Ask a greater number of upfront questions to grey out certain criteria |
|  | Some criteria should be ‘greyed out’ at earlier RIBA stages |
|  | Add initial filter questions to to grey out certain building layers if only certain parts being completed (i.e. shell and core) |
|  | Would be useful to provide a column suggesting the benefits of meeting this criteria (both contextually (this will reduce embodied carbon) and in terms of GLA (e.g. a.3) |
|  | Users should not be allowed to input whether they intend to be basic, partial or full. This should just be reported back following completion |
| Usability | CPD style training should be provided |
|  | Potential to include worked examples (could show a highly and poorly circular building) to address 2 issues at once |
|  | A paywall should be integrated, requiring users to subscribe in order to achieve access to a bank of suppliers/products which will aid in meeting specific criteria |
|  | Potential to attract sponsorship as with LETI |
|  | Include an overall rating to allow clients to set an overall target or aspiration |
|  | The final scores should be provided in a way that relates them to the GLA principles |
|  | Integration as a web tool would be highly beneficial in terms of ease of use and take up |
|  | Use circular output diagram as landing and navigation page |
|  | Increase font size to aid usability |
|  | Should there be a tick to allow the responses from one column (use) to be copied across to another for ease of use? This may have negative impacts with people not reading fully |
|  | The output wheel works well as a ‘badge of honour’ to highlight the success of the project on an output certificate |
|  | Additional information section is really useful |
|  | The tool works well in prompting discussion and debate in areas that would otherwise be disregarded |
|  | An area where additional criteria which have been met but are not listed may be added would be useful |
| General | Timber to be included as a structural type in zonal disaggregation |
|  | Whole life carbon is already required as part of the GLA carbon statement – it being asked here is not unreasonable |
|  | Ask which date a building was designed rather than to which code of practice |
|  | Replace EC building types with ‘domestic, industrial, office, retail’ |
|  | Create a list of who should be sat around the table when filling this in |
|  | Include feedback buttons on various locations within the tool (each page) to allow user to input feedback upon specific elements. This will allow for improvements to be made in the future |

# Test project

Following the implementation of changes from the stakeholder workshop, the final prototype version was tested on a live new-build project at the concept design stage. As outlined in the main body, live and follow-up feedback verified the first three objectives of the tool. Figure 1 shows outputs from the first and second design iterations illustrating initially high achievement in circularity criteria and improvements across three circularity principles in following project meetings. Feedback highlighted that *regenerate* engaged stakeholders across building layers and circularity principles and improved the design solution towards circularity highlighting aspects of circular design that would have otherwise been overlooked, despite some predetermined design decisions limiting the improvement of the deconstructability of building layers.


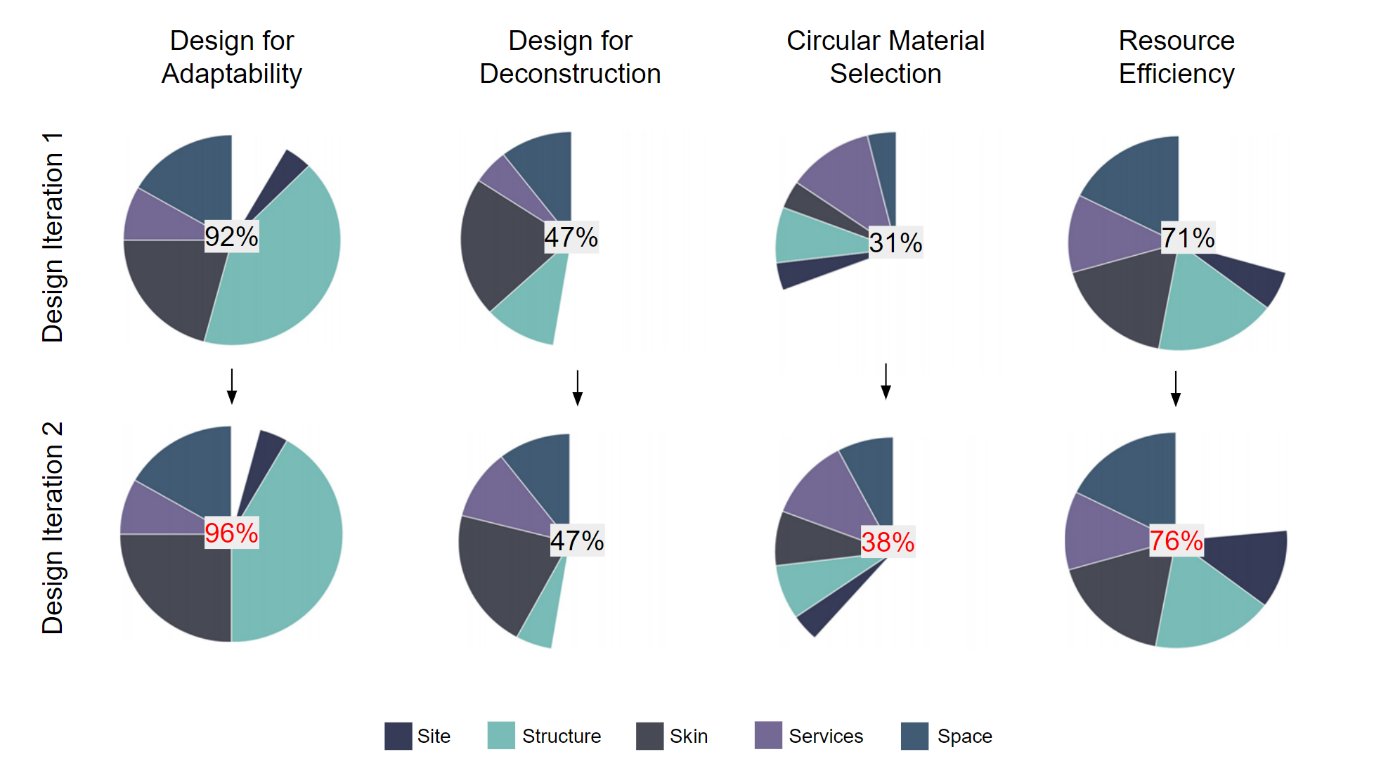


Figure 2: Summary charts showing the percentage achievement of circularity credits across circularity principles for the test project resulting from the initial project meeting (design iteration 1) and the revised project meeting (design iteration 2).

# List of circularity criteria

The following tables list circularity criteria by layer for each circularity principle and are related to the GLA Circular Economy Statement Guidance core principles (presented in section 5).

## Design for adaptability

Table 3: Circularity credits associated with the circularity principle design for adaptability

| Layer | Criteria reference | Criterion | GLA reference |
| --- | --- | --- | --- |
| Site | DfA1 | Capacity of drainage systems suitable for further increase due to climate change | B1 |
|  | DfA2 | Foundations over-sized to accommodate future vertical extensions | B1 |
| Structure | DfA3 | Structural grid allows for flexibility and future change of use | B1 |
|  | DfA4 | Floor loading design criteria allow for flexibility and future change of use | B1 |
|  | DfA5 | Typical floor-to-ceiling height allows for future change of use | B1 |
|  | DfA6 | Circulation capacity sufficient for different uses | B1 |
|  | DfA7 | Structural cores allow for future change of use | B1 |
|  | DfA8 | Period of structural fire resistance appropriate for different uses | B1 |
|  | DfA9 | Fire strategy and means of escape are appropriate for different uses | B1 |
|  | DfA10 | Structure designed to accommodate future expansion of riser space and service voids | B1 |
|  | DfA11 | Serviceability limit state design allows for future change of use | B1 |
|  | DfA12 | Connections and components have high durability | B1 |
| Skin | DfA13 | Façade insulation suitable for different uses/changing climate | B1, A2 |
|  | DfA14 | Sound insulation rating appropriate for different uses | B1 |
|  | DfA15 | Glazing ratios appropriate for different uses | B1 |
|  | DfA16 | Fire rating of façade is appropriate for different uses | B1 |
|  | DfA17 | Connections and components have high durability | B1 |
|  | DfA18 | Environmental design strategies allow for future change of use | B1, A2 |
| Services | DfA19 | Services are easily upgradable for future change of use / changing performance requirements | B1 |
|  | DfA20 | Horizontal and vertical service risers allow for ease of mechanical service upgrades, capacity expansion, and adaptation for change of use | B1 |
| Space | DfA21 | Circulation capacity is sufficient for different uses | B1 |
|  | DfA22 | Fire strategy and means of escape are appropriate for different uses | B1 |
|  | DfA23 | Acoustic performance appropriate for different uses / easily adaptable to changing requirements | B1 |
|  | DfA24 | Internal partitions are demountable to enable flexibility of use | B1 |

## Design for deconstruction

Table 4: Circularity credits associated with the circularity principle design for deconstruction

| Layer | Criteria reference | Criterion | GLA reference |
| --- | --- | --- | --- |
| Site | DfD1 | Foundation design is considered to be reversible | B1 |
| Structure | DfD2 | Reversible, mechanical connections are used instead of chemical alternatives | B1 |
|  | DfD3 | Access to structural connections provided to allow for ease of deconstruction and a high percentage recovery of material | B1 |
|  | DfD4 | The number of different types of structural connections are minimized | B1 |
|  | DfD5 | A deconstruction plan has been produced | B1 |
|  | DfD6 | Material passport style inventory recorded | B1 |
| Skin | DfD7 | Reversible, mechanical connections are used instead of chemical alternatives | B1 |
|  | DfD8 | Access to façade connections provided to allow for ease of dismount and ensure high percentage recovery of material | B1 |
|  | DfD9 | Number of different types of connections to façades minimized | B1 |
|  | DfD10 | Façade replacement or upgrade strategy in place | B1 |
|  | DfD11 | Material passport style inventory recorded | B1 |
| Services | DfD12 | Reversible, mechanical connections are used instead of chemical alternatives | B1 |
|  | DfD13 | Access to services is provided to allow for ease of deconstruction, upgrade and a high percentage recovery of material | B1 |
|  | DfD14 | Number of different types of connections to services minimized | B1 |
|  | DfD15 | Material passport style inventory recorded | B1 |
| Space | DfD16 | Reversible, mechanical connections are used instead of chemical alternatives | B1 |
|  | DfD17 | Access to internal connections / fixings provided to allow for ease of deconstruction and ensure high percentage recovery of material | B1 |
|  | DfD18 | Number of different types of connections to internal spaces minimized | B1 |
|  | DfD19 | Material passport style inventory recorded | B1 |

## Circular materials selection

Table 5: Circularity credits associated with the circularity principle circular materials selection

| Layer | Criteria reference | Criterion | GLA reference |
| --- | --- | --- | --- |
| Site | CMS1 | Where demolition / deconstruction of (part of) an existing structure is taking place, has a pre-demolition audit been completed? (if no demolition / deconstruction is taking place, respond 'Yes') |  |
| Structure | CMS2 | Materials, components and products sourced as part of a leasing / buy-back scheme | A3 |
|  | CMS3 | Materials, components and products registered with a reclamation database | B1, A1, A3 |
|  | CMS4 | Use of toxic or hazardous materials and coatings is minimised | A2 |
|  | CMS5 | Biological materials remain uncontaminated to allow for future return to nature | A2 |
|  | CMS6 | Composition of structural elements ensures future reusability |  |
|  | CMS7 | Material ingredient lists declared (including finishing material such as paints) | B1, A1, A3 |
|  | CMS8 | The use of secondary finishes has been avoided | B1 |
| Skin | CMS9 | Materials, components and products sourced as part of a leasing / buy-back scheme | A3 |
|  | CMS10 | Materials, components and products registered with a reclamation database | B1, A1, A3 |
|  | CMS11 | Use of toxic or hazardous materials and coatings is minimised | A2 |
|  | CMS12 | Biological materials remain uncontaminated to allow for future return to nature | A2 |
|  | CMS13 | Material ingredient lists declared (including finishing material such as paints) | B1, A1, A3 |
|  | CMS14 | The use of secondary finishes has been avoided | B1 |
| Service | CMS15 | Materials, components and products sourced as part of a leasing / buy-back scheme | A3 |
|  | CMS16 | Materials, components and products registered with a reclamation database | B1, A1, A3 |
|  | CMS17 | Use of toxic or hazardous materials and coatings is minimised | A2 |
|  | CMS18 | Biological materials remain uncontaminated to allow for future return to nature | A2 |
|  | CMS19 | Material ingredient lists declared (including finishing material such as paints) | B1, A1, A3 |
|  | CMS20 | The use of secondary finishes has been avoided | B1 |
| Space | CMS21 | Materials, components and products sourced as part of a leasing / buy-back scheme | A3 |
|  | CMS22 | Materials, components and products registered with a reclamation database | B1, A1, A3 |
|  | CMS23 | Use of toxic or hazardous materials and coatings is minimised | A2 |
|  | CMS24 | Biological materials remain uncontaminated to allow for future return to nature | A2 |
|  | CMS25 | Material ingredient lists declared (including finishing material such as paints) | B1, A1, A3 |
|  | CMS26 | The use of secondary finishes has been avoided | B1 |

## 4.4 Resource efficiency

Table 6: Circularity credits associated with the circularity principle resource efficiency

| Layer | Criteria reference | Criterion | GLA reference |
| --- | --- | --- | --- |
| Site | RE1 | Waste' material from on-site buildings or external sources used at its highest value. | A1, A3, B3 (C1 if using demolished elements) |
|  |  | Percentage of material reused from existing on-site buildings or external sources (required as supporting statement) |  |
|  | RE2 | Cut and fill balance achieved | A1 |
|  | RE3 | Temporary works are reusable | A3, C3 |
|  | RE4 | Greywater recycling system incorporated | A3 |
| Structure | RE5 | Waste material from on-site buildings or external sources used at its highest value. | A3, C3 |
|  |  | Percentage of material reused from existing on-site buildings or external sources (required as supporting statement) |  |
|  | RE6 | Considering design requirements (e.g. adaptability criteria), material use is optimized | A1 |
|  | RE7 | Where composite materials/products are used, are these easily separated into component parts for future recycling? (if no composites are used, respond 'Yes') | B1 |
|  | RE8 | Design coordinated to avoid excess cutting and jointing of materials / components that generate waste | A1, A3, C3 |
| Skin | RE9 | Waste material from on-site envelope or external sources used at its highest value. | A3, C3 |
|  |  | Percentage of material reused from existing on-site buildings or external sources (required as supporting statement) |  |
|  | RE10 | Where composite materials/products are used, are these easily separated into component parts for future recycling? (if no composites are used, respond 'Yes') | B1 |
|  | RE11 | Design coordinated to avoid excess cutting and jointing of materials / components | A1, A3, C3 |
| Services | RE12 | Waste material from on-site services or external sources used at its highest value. | A1, A3, C3 |
|  |  | Percentage of material reused from existing on-site buildings or external sources |  |
|  | RE13 | Where composite materials/products are used, are these easily separated into component parts for future recycling? (if no composites are used, respond 'Yes') | B1 |
|  | RE14 | Design coordinated to avoid excess cutting and jointing of materials / components | A1, A3, C3 |
| Space | RE15 | Waste material from on-site envelope or external sources used at its highest value. | A1, A3, B2 (C1 if using demolished elements) |
|  |  | Percentage of material reused from existing on-site buildings or external sources |  |
|  | RE16 | Where composite materials/products are used, are these easily separated into component parts for future recycling? (if no composites are used, respond 'Yes') | B1 |
|  | RE17 | Design coordinated to avoid excess cutting and jointing of materials / components | A1, A3, C3 |

# Greater London Authority Circular Economy Statement Guidance – Core principles

The GLA consider three core principles in their circular economy statement guidance document: 1) Conserve resources and source ethically, 2) Design to eliminate waste, and 3) Manage waste sustainably and at the highest value. As shown in the previous sections, the GLA core principles are related to circularity criteria within the tool to ease the completion of CE statements and compliance with the new London Plan. The definition of these principles is shown in Table 7 for reference alongside circularity criteria.

Table 7: Core principles of the Greater London Authority's Circular Economy Statement Guidance

|  | Conserve resources and source ethically |
| --- | --- |
| A1 | Minimizing the quantity of materials used |
| 2.2.5 | Designs consider opportunities to reduce the demand for building materials, for instance, by prioritizing refurbishment over demolition. Fundamentally, this requires teams to question the design brief and consider whether it can be met by building less and building more efficiently. |
| A2 | Minimizing the quantities of other resources used (fossil fuels, water, land) |
| 2.2.6 | Whilst materials are the primary focus of Circular Economy Statements,  Circular Economy principles also apply to energy, water, land and ecosystems. For example, building on brown field sites rather than virgin land minimizes disruption to the existing landscape, helping to optimize the use of London's limited resources. |
| A3 | Specifying and sourcing materials and other resources responsibly and sustainably |
| 2.2.7 | This principle covers measures such as specifying the use of recycled materials, installing greywater recycling systems, or participating in reuse schemes |
|  | Design to eliminate waste (and for ease of maintenance) |
| B1 | Designing for longevity, adaptability or flexibility and reusability or recoverability |
| 2.2.9 | The design process should include a realistic assessment of the ability of the development to accommodate change, how frequently it will be reconfigured or remodeled, and how to avoid a premature end of life for all components |
| 2.2.10 | Careful decision-making in this regard can add value over the life of the development. For instance, commercial buildings may provide generous floor-to-ceiling heights and open grids to ensure that the interior can accommodate a range of tenants. This flexibility could be enhanced by, for instance, using a relocatable, modular internal partition system that would allow for total reconfiguration without the need to purchase additional materials. |
| B2 | Design out construction, demolition, excavation and municipal waste arising |
| 2.2.11 | Design out waste by considering opportunities for materials optimization, reclamation, and reuse. Minimization of packing, off cuts, damage and rework should be given special attention through off-site, precision manufacture, just-in-time delivery and secure on-site storage. |
| 2.2.12 | When excavating and designing the public realm, topsoil must be given special attention due to its high value and concerns that it is being damaged and wasted at a highly unsustainable rate. Topsoil should never be disposed of to landfill except potentially as a planting medium as part of a site reclamation scheme. Consideration should be given to balancing cut and fill (avoiding any import or export of material) and to techniques that clean and enable reuse of excavation material on site. |
|  | Managing waste sustainably and at the highest value |
| C1 | Managing demolition waste |
| 2.2.16 | In order to manage demolition waste, applicants must consider undertaking an independent pre-demolition audit, implement careful demolition strategies, segregating materials and conducting analysis/monitoring of waste flows to maximize reuse and reclamation. |
| C2 | Managing excavation waste |
| 2.2.17 | Due to the characteristics of this waste stream, not all of it can easily be reused or  recycled, whether on site, locally or otherwise. Nonetheless, excavation material should be put to the best environmental use that is practicable. |
| 2.2.18 | For example, using excavation waste as a resource within the construction of the proposed development in accordance with the Definition of waste Code of Practice (DoWCoP), seeking opportunities for such material to be used in other local construction projects, or other beneficial uses (e.g., quarry restoration) should be prioritized above sending waste to landfill. |
| C3 | Managing construction waste |
| 2.2.19 | Applicants should aim to incorporate measures for managing construction waste  that go above and beyond standard practice where possible |
| 2.2.20 | It is also important to recognize that construction waste arises after the initial construction phase of a development - during the operational phase due to maintenance, refurbishment, and at the end of life. It may be possible to develop plans for managing this waste, based on repair and replacement forecasts or functional adaptability studies. |
| C4 | Managing municipal waste |
| 2.2.21 | This topic should be reflected in the operational waste management plan. Easily accessible space for segregating and storing waste for collection and reuse/recycling/composting must be provided in line with the London Plan and other local authority guidance |
| 2.2.22 | Both domestic and non-domestic developments should explore measures such as consolidated, smart logistics and community-led waste minimization schemes |
